# Effects of Diets Supplemented with Ensiled Mulberry Leaves and Sun-Dried Mulberry Fruit Pomace on the Ruminal Bacterial and Archaeal Community Composition of Finishing Steers

**DOI:** 10.1371/journal.pone.0156836

**Published:** 2016-06-03

**Authors:** Yuhong Niu, Qingxiang Meng, Shengli Li, Liping Ren, Bo Zhou, Thomas Schonewille, Zhenming Zhou

**Affiliations:** 1 State Key Laboratory of Animal Nutrition, Beijing, 100193, P. R. China; 2 College of Animal Science and Technology, China Agricultural University, Beijing, 100193, P. R. China; 3 College of Agronomy and Biotechnology, China Agricultural University, Beijing, 100193, P. R. China; 4 Department of Farm Animal Health, Utrecht University, Utrecht, 3584 CL, The Netherlands; Cleveland Clinic, UNITED STATES

## Abstract

This study investigated the effects of ensiled mulberry leaves (EML) and sun-dried mulberry fruit pomace (SMFP) on the ruminal bacterial and archaeal community composition of finishing steers. Corn grain- and cotton meal-based concentrate was partially replaced with EML or SMFP. The diets had similar crude protein (CP), neutral detergent fiber (NDF), and metabolizable energy. Following the feeding trial, the steers were slaughtered and ruminal liquid samples were collected to study the ruminal microbiome. Extraction of DNA, amplification of the V4 region of the 16S rRNA gene, and Illumina MiSeq pyrosequencing were performed for each sample. Following sequence de-noising, chimera checking, and quality trimming, an average of 209,610 sequences were generated per sample. Quantitative real-time PCR was performed to examine the selected bacterial species in the rumen. Our results showed that the predominant phyla were *Bacteroidetes* (43.90%), *Firmicutes* (39.06%), *Proteobacteria* (4.31%), and *Tenericutes* (2.04%), and the predominant genera included *Prevotella* (13.82%), *Ruminococcus* (2.51%), *Butyrivibrio* (2.38%), and *Succiniclasticum* (2.26%). Compared to the control group, EML and SMFP groups had a higher abundance of total bacteria (*p* < 0.001); however, the bacterial community composition was similar among the three groups. At the phylum level, there were no significant differences in *Firmicutes* (*p* = 0.7932), *Bacteroidetes* (*p* = 0.2330), *Tenericutes* (*p* = 0.2811), or *Proteobacteria* (*p* = 0.0680) levels among the three groups; however, *Fibrobacteres* decreased in EML (*p* = 0.0431). At the genus level, there were no differences in *Prevotella* (*p* = 0.4280), *Ruminococcus* (*p* = 0.2639), *Butyrivibrio* (*p* = 0.4433), or *Succiniclasticum* (*p* = 0.0431) levels among the groups. Additionally, the dietary treatments had no significant effects on the archaeal community composition in the rumen. Therefore, EML and SMFP supplementation had no significant effects on the ruminal bacterial or archaeal community composition of finishing steers.

## Introduction

Mulberry (*Morus* spp., family Moraceae), a fast-growing deciduous tree, thrives under variable climatic conditions ranging from temperate to tropical. Mulberry is a multipurpose tree that produces fruits for human consumption, foliage for rearing silkworm, medicine for patients, and fodder for animal feed [1]. Mulberry leaves are succulent, characterized by high crude protein (CP; 19.4%) and low neutral detergent fiber (NDF; 36.1%) [2]; mulberry fruit pomace is rich in carbohydrates (20.85%), CP (21.86%) and low in NDF (49.06%) [3]. These mulberry by-products represent potential feed sources for herbivores and monogastric animals. Studies have shown that the addition of mulberry leaves to ruminant feed reduces the need for expensive protein supplements [4,5]. Therefore, researchers have evaluated the use of mulberry leaves and fruit pomace in animal feeding [4–9]. Our previous study has shown that ensiled mulberry leaves (EML) and sun-dried mulberry fruit pomace (SMFP) can be used in finishing steer diets without impairing their productive performance or carcass characteristics. Our results revealed that the SMFP-fed group had lower ruminal ammonia and total volatile fatty acid (VFA) concentrations than the EML-fed group [10], probably due to differences in the ruminal microbiome between the two groups. It has been reported that there is a correlation between host physiology and genus abundance. Dietary changes affect the ruminal microbiome (i.e., bacteria, protozoa, and fungi) [11–13], and changes in the ruminal microbiome affect the digestive capacity of the animal (e.g., improved fiber utilization and/or decreased methane production) [14–16].

We hypothesize that the partial replacement of concentrate with 8% EML or 6.3% SMFP in the diet affects the ruminal microbiome. Therefore, the objective of this study was to assess the effects of diets supplemented with EML or SMFP on the ruminal bacterial and archaeal community composition of finishing steers.

## Materials and Methods

Experiments were conducted at the Beef Cattle Research Station of China Agricultural University in Daxing, Beijing. The protocol was approved by the China Agricultural University’s Animal Welfare and Ethical Committee (Permit No. DK1008).

### Animals, diets, and samples

This study was part of a larger experimental trial investigating the effects of EML and SMFP on growth performance, ruminal fermentation, blood biochemical parameters, and carcass characteristics of finishing steers [10]. In which, medium-frame crossbred Simmental steers (357.06 ± 16.5 kg; 15 months of age) were divided into three groups. The control group (CON) received a typical total mixed ration (TMR); the EML group received a typical TMR supplemented with 8% EML; and the SMFP group received a typical TMR supplemented with 6.3% SMFP. Mulberry leaves were harvested from a farm in Daxing district of Beijing, China. The harvested mulberry material was ensiled without additives after chopping. The mulberry silage was then used for the feeding animals after being stored for 50 days. Chemical composition and silage fermentation characteristics of EML or SMFP were shown in our previous report [10]. Mulberry fruit pomace was purchased from a local company (Guosen Co., Beijing, China). For this study, 12 steers, with 4 animals per group, were selected to investigate the effects of EML and SMFP on the ruminal bacterial and archaeal community composition. After slaughtering, ruminal samples (500 ml, consisting of a mixture of liquids and solids) from the dorsal, central, and ventral regions of the rumen were collected, pooled, and strained through four layers of cheesecloth. The resulting liquid samples were stored at -80°C for microflora profiling.

### DNA extraction and pyrosequencing

Metagenomic DNA was extracted from 0.5 g of each homogenized ruminal liquid sample by using a Precellys 24 homogenizer (Bertin Technologies, Montigny-le-Bretonneux, France) plus column method [17]. The rotating speed of the oscillator was set to 5,500 rpm with two circulations at 30 s per circulation. Extracted DNA yield and purity were determined spectrophotometrically in a NanoDrop^TM^ ND-2000 (Thermo Fisher Scientific, Waltham, MA, USA). Genomic DNA samples were diluted to 5 mM with TE buffer. Pyrosequencing was conducted on an on an Illumina MiSeq platform v2 2 × 250 bp paired end protocol yielding paired-end reads. Briefly, DNA was amplified using the universal eubacterial primer set (515f: 5’-GTG CCA GCM GCC GCG GTA A-3’, 806r: 5’-XXX XXX GGA CTA CHV GGG TWT CTA AT-3’), which targets the hypervariable V4 region of the 16S rRNA gene, with the reverse primer containing a 6-bp error-correcting barcode unique to each sample. Amplification was performed with Phusion High-Fidelity PCR Mastermix (New England Biolabs Ltd., Beijing, China) under the following conditions, one cycle at 94°C for 3 min, 30 cycles at 94°C for 45 s, 50°C for 60 s, and 72°C for 90 s, and one cycle at 72°C for 5 min. Amplicons were selected on 2% agarose gels on E-Gel®Size SelectTM Agarose Gel and purified with Agencourt® AMPure® XP Reagent. Purified DNA was quantified with Quant-iTTM Technology (Life Technologies, Inc.) and Quant-iTTM dsDNA Broad-Range Assay Kit. Agilent 2100 BioanalyzerTM with the Agilent High Sensitivity DNA Kit (Agilent Technologies, Inc., Santa Clara, CA) were used to analyze library sizes and molar concentrations.

### Sequence analysis

Sequences were analyzed with the QIIME pipeline. Reads were processed to quality control with Fast QC software. Only sequences without ambiguous characters were included in the analyses. FLASH 1.2.7v software was used to merge paired-end reads from sequencing raw data [18]. Chimeric sequences were removed by using the USEARCH software based on the UCHIME algorithm [19]. To calculate downstream diversity determination (alpha and beta diversity), all samples were subsampled to a size of 100,000 prior to bacterial community comparisons. Microbial diversity was assessed using the QIIME 1.7.0v software [20] with Python scripts. The sequences were clustered into Operational Taxonomic Unit (OTUs) by using de novo OTU picking protocol with a 97% similarity threshold. Taxonomy assignment of OTUs was performed by comparing sequences to the Greengenes database. Alpha diversity analysis (observed species, Chao, and Shannon) were generated. Jackknifed beta diversity included both unweighted and weighted Unifrac distances, and these distances were visualized by Principal Coordinate Analysis (PCoA)[21].

### Quantitative real-time PCR

Quantitative real-time PCR was performed to investigate the relative abundance of microorganisms per gram of homogenized ruminal liquid sample, and the using primers were shown in S5 Table. The standards used for the qPCR amplifications have been used for ruminal bacteria, fungi, protozoa, and methanogenic archaea [22,23]. The average values of relative population size (RPS) for each selected bacterial strain, based on 16S rRNA gene copy number, was calculated as previously reported [24,25]. Selected methanogen copy numbers were estimated by a nested PCR approach [26]. Real-time PCR was performed in an Applied Biosystems StepOne Plus sequence detection system. The 20-μl reaction mixture consisted of 10 μl of 2×SYBR Green Master Mix (Tiangen, Beijing, China), 1 μl of each primer (10 μM working concentration), 6 μl of nuclease-free water, and 2 μl of template containing 10 ng DNA. The PCR conditions consisted of an initial denaturation step at 95°C for 10 min, followed by 40 cycles at 95°C for 10 s, 60°C for 15 s, 72°C for 1 min, and a final extension step at 72°C for 6 min. To determine primer specificity, a melting curve was generated by heating the reaction mixture from 60 to 95°C at 1°C/s, with fluorescence readings at 1°C intervals [27]. Real-time PCR was performed in triplicate for the standards and metagenomic DNA samples. PCR products were confirmed by 1.5% agarose gel electrophoresis. The abundance of ruminal microbes was recorded and multiplied by the dilution factor to determine the total number of target microbe per gram (wet weight).

### Statistical analyses

Read number, sample coverage, unique OTUs, sample richness, and sample diversity were compared using the generalized linear model (GLM) procedure of SAS (version 9.1.3; SAS Institute Inc., Cary, NC, USA). Abundance of phylum and genus was determined to assess the effects of EML or SMFP supplementation. Absolute abundance of microbes was expressed as copies of 18S or16S rRNA genes per gram (wet weight); relative abundance of specific bacterial strains was expressed as the average RPS value. Means were separated using Student-Newman-Keuls test (SNK). Statistical significance was set at *p* ≤ 0.05. Only phylum and genus detected in all rumen samples were defined as shared taxa in the data analyses.

## Results

### Abundance of microbes in the rumen

**Fig 1:**
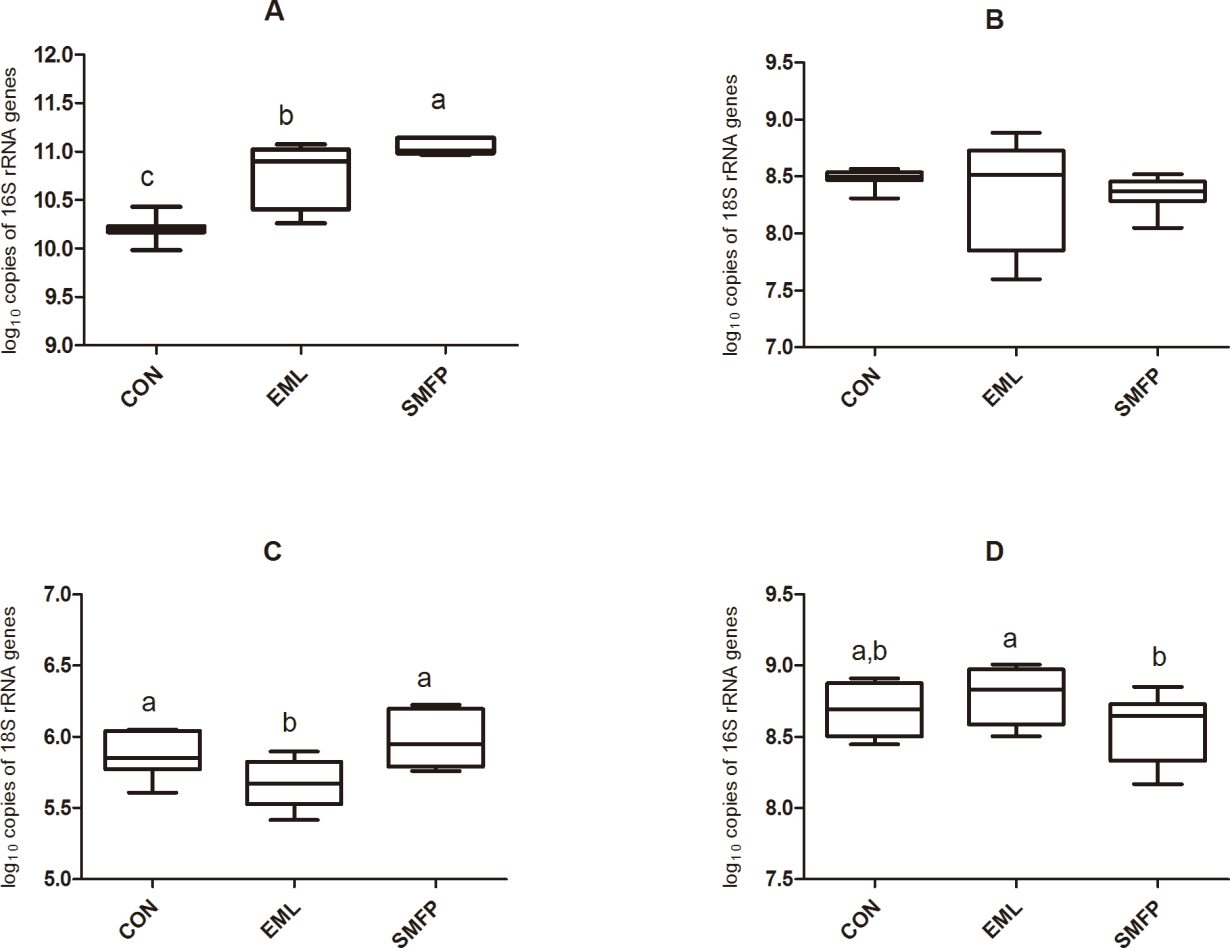
Abundance of microflora in the rumen of finishing steers when corn grain- and cotton seed meal-based concentrate was partially replaced with EML or SMFP. (A), abundance of total bacteria in the rumen; (B), abundance of fungi in the rumen; (C), abundance of protozoa in the rumen; (D), abundance of archaea in the rumen. The boxes represent the interquartile range (IQR) between the first and third quartiles (25th and 75th percentiles, respectively). The horizontal line inside the box represents the median. Whiskers represent the lowest and highest values within 1.5 times the IQR from the first and third quartiles, respectively. Different letters represent significant differences (*p* < 0.05). CON: control group (n = 4); EML: ensiled mulberry leaves group (n = 4); SMFP: sun-dried mulberry fruit pomace group (n = 4).

Fig 1A shows the abundance of total bacteria per gram (wet weight) in the rumen. EML and SMFP groups had a higher abundance of total bacteria than the CON group (*p* < 0.001). There were no significant differences in the abundance of fungi among EML, SMFP, and CON groups (Fig 1B). Fig 1C shows the abundance of ruminal protozoa per gram (wet weight). The protozoa abundance in the SMFP group was higher than that in the EML group (*p* = 0.0089). There was no significant treatment-effect on the abundance of ruminal protozoa per gram compared with the CON group. The abundance of archaea per gram is shown in Fig 1D. The archaeal abundance in the SMFP group was lower than that in the EML group (*p* = 0.0302). In contrast, the abundance of archaea in the EML group was similar to that obtained in the CON group. The abundance of archaea, expressed as 16S rRNA gene copies per gram, was 5.30×10^8^ in EML, 6.85×10^8^ in SMFP, and 4.29×10^8^ in CON.

### Sequencing and general ruminal community composition

We generated 2,712,307 raw sequences, of which 2,515,323 passed all filtering metrics with an average length of 292 bp. All individual sequencing and coverage metrics are presented in S1 Table. Individual sample sequence counts ranged from 165,804 to 257,130 sequences, with an average of 209,610 sequences. Following OTU picking and chimera checking, a total of 24,028 OTUs were calculated for 12 samples at 3% dissimilarity. Each sample had an average of 9,693 OTUs. Following normalization to 100,000 reads, richness estimates and diversity indices were generated (Table 1). Good’s coverage for each sample ranged from 0.9657 to 0.9723, with an average of 0.9680. The average Simpon’s diversity was 99.70.

**Table 1:** Unique OTUs, richness estimates, and diversity indices in ruminal samples from each dietary group.

	Experimental diet^1^				
Item	CON	EML	SMFP	SEM^2^	*p* value
SeqsNum	221929.75	205299.50	201601.50	12626.81	0.5060
OTUsNum	9751.00	9927.00	9403.50	476.77	0.7395
EvenSeqsNum	100000	100000	100000	-	-
EvenOTUsNum	6784.00	7094.50	6777.50	227.68	0.5530
ACE	12466.86	12983.51	12523.74	536.82	0.7632
Simpson	1.00	1.00	1.00	0.00	0.7099
Shannon	10.12	10.21	10.11	0.08	0.5978
PD_whole_tree	263.10	270.61	261.91	7.22	0.6651
chao1	12578.87	13156.99	12803.46	505.06	0.7252
observed_species	6784.00	7094.50	6777.50	227.68	0.5530
goods_coverage (%)	96.86	96.71	96.84	0.02	0.7160

^1^CON: control group (n = 4); EML: ensiled mulberry leaves group (n = 4); SMFP: sun-dried mulberry fruit pomace group (n = 4).

^2^SEM: standard error of the mean.

**Fig 2:**
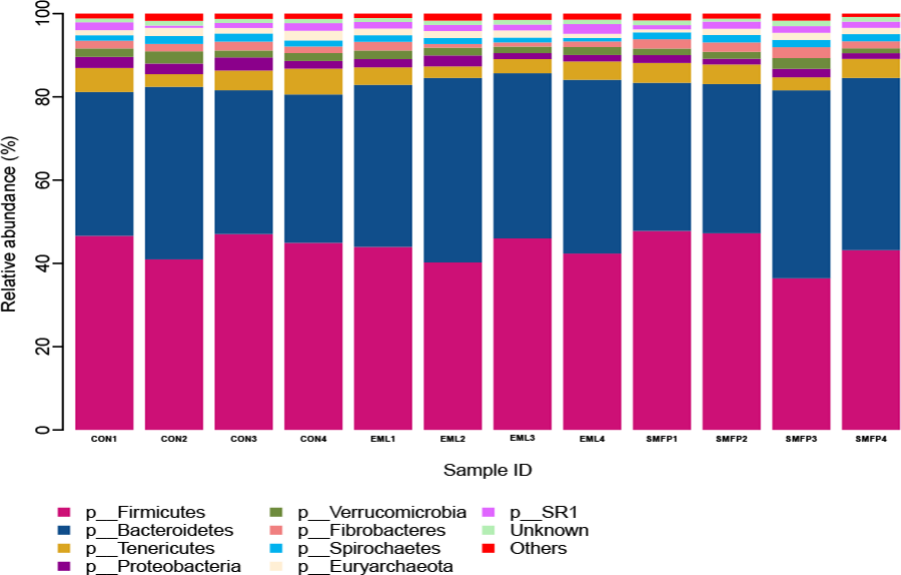
Phylum level composition. Stacked bar plot showing the phylum-level composition for individual steer rumen sample. CON: control group (n = 4); EML: ensiled mulberry leaves group (n = 4); SMFP: sun-dried mulberry fruit pomace group (n = 4).

The most abundant phyla were *Bacteroidetes* (43.90%) and *Firmicutes* (39.06%), followed by *Proteobacteria* (4.31%) and *Tenericutes* (2.04%) in all samples. Minor phyla included *Verrucomicrobia* (1.91%), *Fibrobacteres* (1.79%), *Spirochaetes* (1.54%), and SR1 (1.50%). The other known phylum accounted for 1.37%; unclassified sequences were 1.05% of the total sequences. Fig 2 and S2 Table show the community composition in individual samples. In the stacked bar chart, each bar represents the average relative abundance of bacterial phylum. The top nine phyla with high relative abundance are presented. These phyla comprised 97.58% of the reads. The most abundant genera were *Prevotella* (13.82%), *Ruminococcus* (2.51%), *Butyrivibrio* (2.38%), and *Succiniclasticum* (2.26%). Minor genera such as *Fibrobacter*, YRC22, CF231, *Coprococcus*, *Treponema*,*Clostridium*, *Anaerovibrio*, and *Acetobacter* accounted for 1.79%, 1.64%, 1.41%, 1.30%, 1.14%, 0.82%, 0.70%, and 0.27%, respectively. The other known genus accounted for 3.90%; 64.65% of the sequences were unclassified at the genus level. S3 Table shows the percent abundance of genera in all ruminal samples.

### Effects of dietary treatment on bacterial community

**Fig 3:**
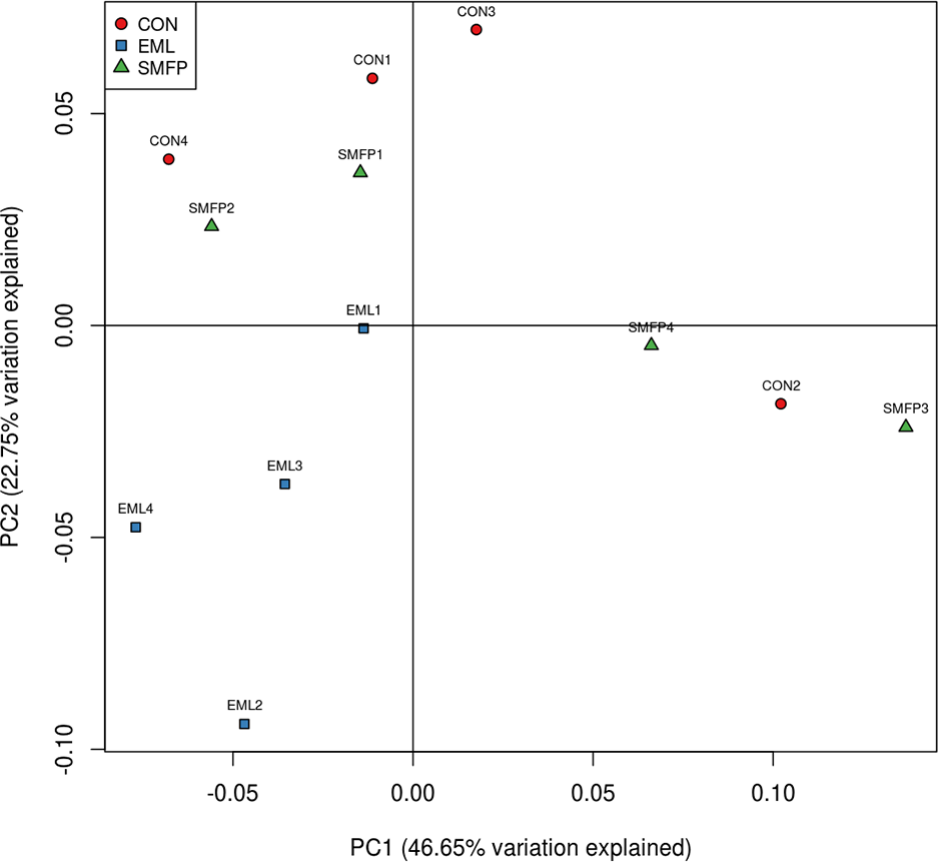
Principal Coordinate Analysis (PCoA) of bacterial community structures of the ruminal microbiome in CON (red circles), EML (blue squares), and SMFP (green triangles) groups. PCoA plots were constructed using the weighted UniFrac method. CON: control group (n = 4); EML: ensiled mulberry leaves group (n = 4); SMFP: sun-dried mulberry fruit pomace group (n = 4).

Table 1 shows the unique OTUs, richness estimates, and diversity indices in ruminal samples obtained from EML, SMFP, and CON groups. There were no significant differences in the sequence sets among the three groups (*p* > 0.50). Thermal double dendrograms of the most abundant top 50 bacterial OTUs demonstrated that samples could not be clearly grouped in the same treatment (S1 Fig and S4 Table), which suggests that the bacterial communities of different treatments were substantially similar. PCoA based on a weighted UniFrac metric was performed to compare all samples. The dividing line was less obvious, and the two principal components explained 21.72% of the variation. The CON group samples could not be distinguished from those of EML or SMFP (Fig 3).

The relative abundance of all taxa in the samples was used to describe the impact of the dietary treatments on the ruminal bacterial composition. At the phylum level, except for a reduction of *Fibrobacteres* in the EML group (*p* = 0.0431), there were no significant differences in ruminal bacterial composition among EML, SMFP, and CON (Table 2); therefore, there was a dominant profile common to many of the finishing steers. Compared to CON, EML and SMFP had reduced *Proteobacteria* (*p* = 0.0680) and *Spirochaetes* (*p* = 0.0696). At the genus level, there were no significant differences among the three groups (Table 3). Even though *Fibrobacter* increased in the SMFP group (*p* = 0.0431) and *Treponema* decreased in the EML group (*p* = 0.0388), these differences were modest (< 1-fold). These findings suggest that the microbial community was not significantly different among the three groups.

**Table 2:** Effect of dietary treatments on the phyla (as a percentage of the total sequences) of the ruminal bacterial community.

	Experimental diet^1^				
Phylum	CON	EML	SMFP	SEM^2^	*p* value
*Firmicutes*	44.90	43.14	44.02	1.85	0.7932
*Bacteroidetes*	36.54	41.16	38.85	1.78	0.2330
*Tenericutes*	4.94	3.69	4.32	0.51	0.2811
*Proteobacteria*	2.55	1.90	2.22	0.24	0.0680
*Verrucomicrobia*	2.14	1.86	2.00	0.24	0.5362
*Fibrobacteres*	1.84^a^	1.33^b^	1.58^a,b^	0.20	0.0431
*Spirochaetes*	1.65	1.26	1.45	0.13	0.0696
*Euryarchaeota*	1.71	1.50	1.60	0.22	0.5774
*SR1*	1.37	1.69	1.53	0.24	0.6296
Others	1.36	1.43	1.39	0.16	0.9078
Unknown	1.01	1.04	1.03	0.08	0.8544

^1^CON: control group (n = 4); EML: ensiled mulberry leaves group (n = 4); SMFP: sun-dried mulberry fruit pomace group (n = 4). Relative sequence abundance (%).

^2^SEM: standard error of the mean.

Different letters in a row represent significant differences (*p* < 0.05).

**Table 3:** Effect of dietary treatments on the genera (as a percentage of the total sequences) of the ruminal bacterial community.

	Experimental diet^1^				
Genus	CON	EML	SMFP	SEM^2^	*p* value
*Prevotella*	13.34	12.26	15.87	1.92	0.4280
*Ruminococcus*	2.52	2.30	2.72	0.17	0.2639
*Butyrivibrio*	2.19	2.35	2.61	0.23	0.4433
*Succiniclasticum*	2.70	1.93	2.15	0.21	0.0680
*Fibrobacter*	1.84^a,b^	1.33^b^	2.20^a^	0.20	0.0431
*YRC22*	1.59	1.28	2.05	0.20	0.0628
*Treponema*	1.55^a^	1.08^b^	1.61^a^	0.13	0.0388
*CF231*	1.17	1.40	1.34	0.09	0.2503
*Coprococcus*	1.15	0.94	1.33	0.10	0.0612
*Clostridium*	0.81	0.74	0.90	0.10	0.5492
*Anaerovibrio*	0.84	0.51	0.75	0.15	0.3103
*Acetobacter*	0.65	0.05	0.12	0.20	0.1257
Others	4.10	3.88	3.72	0.16	0.2733
Unknown	63.97	68.62	61.37	2.23	0.1196

^1^CON: control group (n = 4); EML: ensiled mulberry leaves group (n = 4); SMFP: sun-dried mulberry fruit pomace group (n = 4). Relative sequence abundance (%).

^2^SEM: standard error of the mean.

Different letters in a row represent significant differences (*p* < 0.05).

### Proportion of selected bacteria and archaea in the rumen

**Fig 4:**
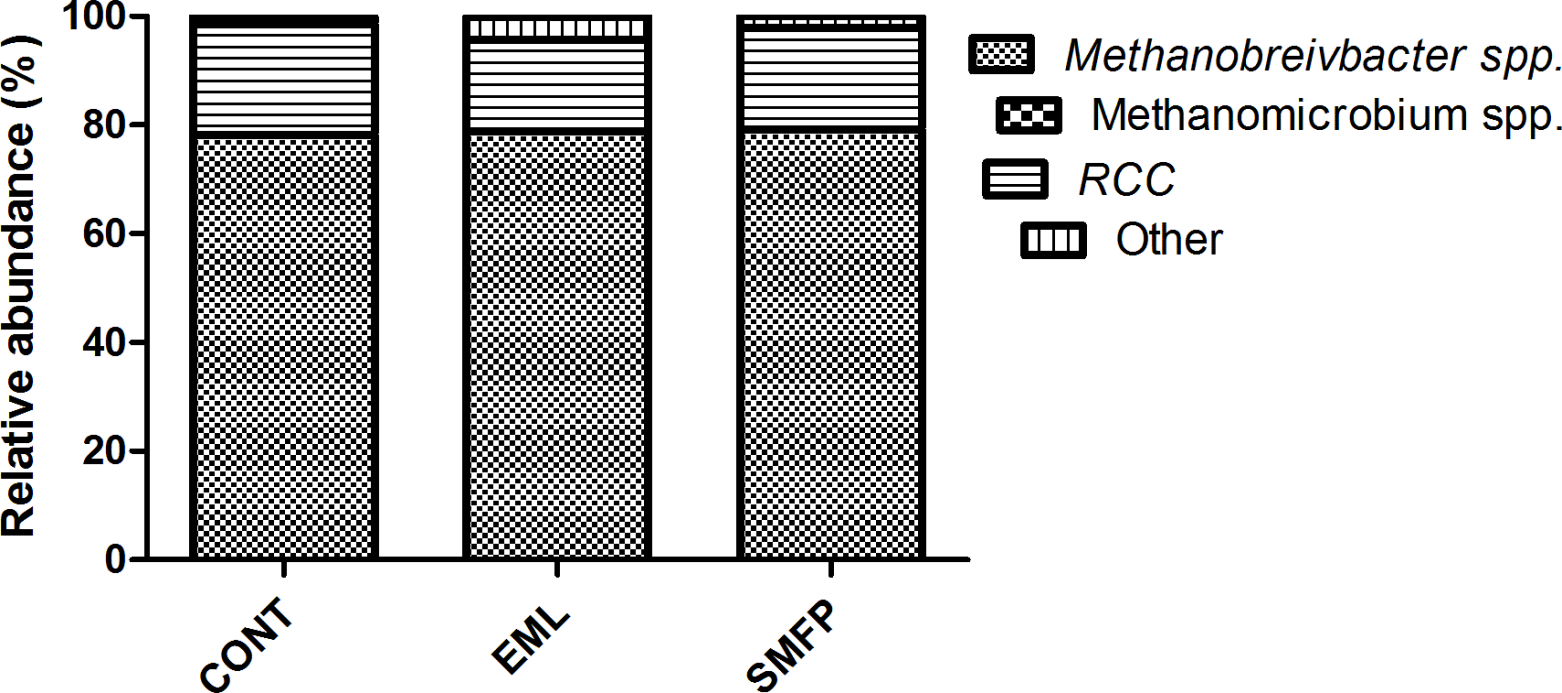
Distribution of different archaeal taxa in the rumen of finishing steers when corn grain- and cotton seed meal-based concentrate was partially replaced with EML or SMFP. Methanogen 16S rRNA gene was amplified using universal archaeal primer sets and quantified using nested PCR with taxon-specific primer sets for *Methanobrevibacter* spp., RCC, and *Methanomicrobium* spp. CON: control group (n = 4); EML: ensiled mulberry leaves group (n = 4); SMFP: sun-dried mulberry fruit pomace group (n = 4).

The effects of EML and SMFP on selected ruminal bacteria are shown in Table 4. There were significant differences among the groups with respect to RPS of several species. Thirteen examined species contributed to < 15% of the total domain bacteria. EML and SMFP affected the composition of ruminal bacteria, as reflected by the specific species. In EML and SMFP, the proportion of *Eubacterium ruminatium*, *Megasphaera elsdenii*, *Ruminococcus albus*, and *Streptococcus bovis* decreased (*p* = 0.0058, *p* = 0.0106, *p* = 0.0179, and *p* = 0.0282, respectively) relative to that present in CON. On the other hand, the proportion of *Ruminococcus flavefaciens* was not significantly different among the three groups. Similarly, there were no significant differences in the RPS of *Prevotella ruminicola*, *P*. *brevis*, and *P*. *bryantii* (*p* = 0.1367, *p* = 0.2162, and *p* = 0.1768, respectively) among the groups. The RPS of the dextrin-fermenting *Succinivibrio dextrinisolvens* was similar to that of *Ruminobacter amylophilus* and *Selenomonas ruminantium*. Additionally, dietary treatments had no significant effects on the archaeal community composition of the rumen (Fig 4). *Methanobrevibacter* spp. and RCC were proportionally more abundant in the rumen (98% of the 16s rRNA gene).

**Table 4:** Ruminal bacterial abundance in finishing steers fed a total mixed ration supplemented with ensiled mulberry leaves (EML) or sun-dried mulberry fruit pomace (SMFP).

	Experimental diet^1^				
Bacteria	CON	EML	SMFP	SEM^2^	*p* value
*Fibrobacter succinogenes*	0.5495^a^	0.2669^b^	0.4308^a^	0.0472	0.0154
*Prevotella bryantii*	2.9567	2.8007	4.3412	0.5537	0.1768
*Prevotella ruminicola*	4.4930	4.7026	7.0391	0.8410	0.1367
*Ruminococcus flavefaciens*	1.0488	0.9010	1.0716	0.1998	0.8125
*Selenomonas ruminantium*	1.9291	1.4998	1.3699	0.2791	0.3918
*Succinivibrio dextrinosolvens*	0.0321	0.0411	0.0033	0.0206	0.4479
*Butyrivibrio fibrisolvens*	0.1006	0.0034	0.0009	0.0467	0.2998
*Eubacterium ruminatium*	0.1416^a^	0.0385^b^	0.0130^b^	0.0184	0.0058
*Megasphaera elsdenii*	0.0012^a^	0.0003^b^	0.0001^b^	0.0002	0.0106
*Prevotella brevis*	0.3562	0.3130	0.0561	0.1148	0.2162
*Ruminobacter amylophilus*	0.0042	0.0345	0.0005	0.0199	0.4635
*Ruminococcus albus*	0.0320^a^	0.0107^b^	0.0044^b^	0.0050	0.0179
*Streptococcus bovis*	0.0074^a^	0.0026^b^	0.0003^b^	0.0014	0.0282

^1^CON: control group (n = 4); EML: ensiled mulberry leaves group (n = 4); SMFP: sun-dried mulberry fruit pomace group (n = 4). Population sizes are expressed as percentages of the 16S rRNA gene copy number of the total bacteria (%).

^2^SEM: standard error of the mean.

Different letters in a row represent significant differences (*p* < 0.05).

## Discussion

Microbes form a complex network in the rumen and ferment the fibrous plant material ingested by the ruminant. Fermentation end-products, such as acetate, propionate, and other short-chain fatty acids, are absorbed by the host across the rumen wall. There is a correlation between host physiology and microbial abundance [11]. Our previous report showed that EML or SMFP had no effects on animal performance. Significant differences in ruminal fermentation might be attributed to changes in the microbial population. Our study findings revealed that the abundance of ruminal bacteria tended to increase in the EML and SMFP groups. The presence of higher levels of digestible carbohydrates and/or total soluble solids (especially glucose and fructose) in EML and SMFP may have contributed to a higher number of ruminal bacteria, which probably resulted in differences in VFA concentrations. Our results showed that archaeal abundance represented approximately 1% of the total prokaryotic (bacteria and archaea) populations, consistent with past findings [25,28]. The abundance of archaea decreased when the diet was supplemented with SMFP. According to previous reports, bioactive secondary metabolites in plants inhibit methane production in the rumen [29]. Mulberry fruits are rich in phenolics, anthocyanins, and flavonoids [6]; processed mulberry pomace inhibits methanogen activity. Additionally, the abundance of archaea obtained in our study was higher than that reported in lactating (4.73×10^7^ cells/g wet weight) and non-lactating dairy cows (5.58×10^6^ cells/g wet weight) [30,31], similar to that reported in cows (1.9×10^8^ to 3.31×10^8^ cells/ml) [32] and sheep (6.17×10^8^ cells/ml) [28], and lower than that reported in steers (1.34×10^9^ cells/ml) [23]. Therefore, variability in the abundance of ruminal archaea could be attributed to differences in experimental methodologies, ruminant species, geographical location, and diets. Even though the abundance of bacteria and archaea changed, the abundance of fungi and protozoa remained constant during the experiment. The limited amounts of EML and SMFP may have contributed to these results.

Consistent with previous reports [33–39], *Bacteroidetes* and *Firmicutes* were the most abundant phyla, followed by *Proteobacteria*, *Tenericutes*, and *Verrucomicrobia*. EML and SMFP supplementation had no effects on the predominant phyla in the rumen, except on *Fibrobacteres*. Within the phylum *Bacteroidetes*, *Prevotella* represented the most abundant genus in the rumen. *Prevotella* is predominant in both forage-fed and grain-fed livestock [12,40] and comprises a large part of the genetic and metabolic diversity in ruminal microbial communities [41], independent of geographical location or management system. The treatments had no effects on the relative abundance of selected *Prevotella* species, similar to previous findings [25]. Three cellulolytic species (*Prevotella bryantii*, *P*. *ruminicola*, and *P*. *brevis*) represented approximately 1% of the total bacterial 16S rRNA gene copies. Such a small cellulolytic population suggests that a significant fraction of ruminal cellulose degradation may be due to a combination of cellulolytic eukaryotes and uncultured cellulolytic bacterial species.

The sequencing results revealed a higher abundance of phylum *Fibrobacteres* and genus *Fibrobacter* in CON than in EML or SMFP. Even though *Fibrobacteres* represented a very small fraction, its presence in the samples suggests that *Fibrobacteres* occupies a special ecological niche in the rumen. In fact, the genus *Fibrobacter* is involved in readily digested plant-based cellulose in ruminant animals. *F*. *succinogenes* is one of the two most cultivated species in its phylum and one of the most highly cellulolytic species [42]. Previous phylogenetic analyses of ruminal microbiomes have shown that all *Fibrobacteres* sequences belonged to the genus *Fibrobacter*, and 45% of sequences were assigned to *F*. *succinogenes* [43]. In our study, the average abundance of *F*. *succinogenes* was approximately 23.23% within the genus *Fibrobacteres*. The experimental diets and animal species may be responsible for this difference. Complete genome sequence analyses revealed that a high number of genes coded for glycoside hydrolases, carbohydrate-binding modules, carbohydrate esterases, and polysaccharide lyases [44]. The abundance of *F*. *succinogenes* should be similar between the groups because of their similar fiber levels; however, the results obtained were the opposite. Compared to corn, mulberry leaves or residues contain more fructose and pectin and less starch. In our study, the three groups had similar NDF levels; the carbohydrate structure was probably responsible for the differences in microbial community composition.

The genus *Treponema* is commonly present in the rumen. Species of this genus are involved in the degradation of soluble fibers. *T*. *bryantii* forms a symbiotic relationship with *F*. *succinogenes* strains in the rumen [45], and *T*. *saccharophilum* is a pectinolytic bacterium [46,47]. Compared with EML, the SMFP group had higher abundance of the genus *Treponema* probably due to its higher pectin content.

In the rumen, archaea consist of strictly anaerobic methanogens that belong to one of these three genera: *Methanobrevibacter*, *Methanomicrobium*, and rumen cluster C (RCC) [48]. Our findings showed that EML and SMFP supplementation affected the overall archaeal community composition in the rumen. This was evident because the abundance of *Methanobrevibacter* increased in EML and SMFP. *Methanobrevibacter* is considered to be the most predominant methanogen in the rumen of cattle, dairy cows, and sheep [30,31,49–52]. The proportion of *Methanobrevibacter* was approximately 78–79%. However, a recent report detected higher proportions of RCC (72%) and lower proportions of *Methanomicrobiumclade* (19%). The authors have proposed that primers that result in non-specific amplification products might have affected the analyses [26]. With the exception of primers, host factors, diets, genetic variations, experimental methodologies, and geographical location may have contributed to the differences in the results.

The results of this study revealed that the partial replacement of corn grain and cotton seed meal with EML or SMFP had no substantial effects on the ruminal microflora composition. Microbes form a complex network in the ruminal ecosystem and ferment the fibrous plant material ingested by the ruminant. Further studies are required to determine whether other fungi and protozoa species are associated with fermentation processes in the rumen.

## Supporting Information

S1 Fig Effect of diets containing EML or SMFP on ruminal bacterial composition of finishing steers for the top 50 most abundant OTUs. CON: control group (n = 4); EML: ensiled mulberry leaves group (n = 4); SMFP: sun-dried mulberry fruit pomace group (n = 4).
(TIF)

S1 Table Individual finishing bulls for unique OTUs, richness estimates, and diversity indices within the rumen content. (DOCX)

S2 Table Relative abundance (%) of phylum in ruminal sample of individual finishing steers. (DOCX)

S3 Table Relative abundance (%) of shared genus in ruminal sample of individual finishing steers. (DOCX)

S4 Table Top 50 most abundant ruminal bacterial OTUs in individual finishing steers. (XLSX)

S5 Table Primers used in real-time PCR for the detection of ruminal archaeal and bacterial species/taxa. (DOCX)

## References

[1] Sharma SK, Zote KK (2010) MULBERRY—A multi purpose tree species for varied climate. Range Management and Agroforestry 31: 97–101.

[2] Sahoo A, Singh B, Sharma OP (2011) Evaluation of feeding value of Eupatorium adenophorum in combination with mulberry leaves. Livestock Science 136: 175–183.

[3] Zhou B, Meng QX, Ren LP, Shi FH, Wei Z, Zhou ZM (2012) Evaluation of chemical composition, in situ degradability and in vitro gas production of ensiled and sun-dried mulberry pomace. Journal of Animal and Feed Sciences 21: 188–197.

[4] Salinas-Chavira J, Castillo-Martinez O, Ramirez-Bribiesca JE, Mellado M (2011) Effect of increasing levels of white mulberry leaves (Morus alba) on ruminal dry matter degradability in lambs. Tropical Animal Health and Production 43: 995–999. 10.1007/s11250-011-9797-1 21336982

[5] Vu CC, Verstegen MWA, Hendriks WH, Pham KC (2011) The Nutritive Value of Mulberry Leaves (Morus alba) and Partial Replacement of Cotton Seed in Rations on the Performance of Growing Vietnamese Cattle. Asian-Australasian Journal of Animal Sciences 24: 1233–1242.

[6] Cheong SH, Kim KH, Jeon BT, Park PJ, Hwang IH, Choi NJ, et al (2012) Effect of mulberry silage supplementation during late fattening stage of Hanwoo (Bos taurus coreanae) steer on antioxidative enzyme activity within the longissimus muscle. Animal Production Science 52: 240–247.

[7] Todaro M, Sinacori A, Marinaro G, Alicata ML, Giaccone P (2007) Palatability and in vivo digestibility of mulberry leaves (Morus latifolia CV. Kokusou 21) in sheep feeding. Journal of Animal and Veterinary Advances 6: 509–512.

[8] Liu JX, Yao J, Yan B, Yu JQ, Shi ZQ (2001) Effects of mulberry leaves to replace rapeseed meal on performance of sheep feeding on ammoniated rice straw diet. Small Ruminant Research 39: 131–136. 11182305 10.1016/s0921-4488(00)00180-2

[9] Doran MP, Laca EA, Sainz RD (2007) Total tract and rumen digestibility of mulberry foliage (Morus alba), alfalfa hay and oat hay in sheep. Animal Feed Science and Technology 138: 239–253.

[10] Zhou Z, Zhou B, Ren L, Meng Q (2014) Effect of ensiled mulberry leaves and sun-dried mulberry fruit pomace on finishing steer growth performance, blood biochemical parameters, and carcass characteristics. PLoS One 9: e85406 10.1371/journal.pone.0085406 24427304 PMC3888424

[11] Jami E, White BA, Mizrahi I (2014) Potential role of the bovine rumen microbiome in modulating milk composition and feed efficiency. PLoS One 9: e85423 10.1371/journal.pone.0085423 24465556 PMC3899005

[12] Pitta DW, Pinchak WE, Dowd S, Dorton K, Yoon I, Min BR, et al (2014) Longitudinal shifts in bacterial diversity and fermentation pattern in the rumen of steers grazing wheat pasture. Anaerobe 30: 11–17. 10.1016/j.anaerobe.2014.07.008 25086244

[13] Henderson G, Cox F, Ganesh S, Jonker A, Young W, Janssen PH (2015) Rumen microbial community composition varies with diet and host, but a core microbiome is found across a wide geographical range. Sci Rep 5: 14567 10.1038/srep14567 26449758 PMC4598811

[14] Weimer PJ (2015) Redundancy, resilience and host specificity of the ruminal microbiota: Implications for engineering improved ruminal fermentations. Frontiers in Microbiology 6. 10.3389/fmicb.2015.00296 PMC4392294 25914693

[15] Mao SY, Huo WJ, Zhu WY (2014) Microbiome-metabolome analysis reveals unhealthy alterations in the composition and metabolism of ruminal microbiota with increasing dietary grain in a goat model. Environ Microbiol. 10.1111/1462-2920.12724 25471302

[16] Ding G, Chang Y, Zhao L, Zhou Z, Ren L, Meng Q (2014) Effect of Saccharomyces cerevisiae on alfalfa nutrient degradation characteristics and rumen microbial populations of steers fed diets with different concentrate-to-forage ratios. J Anim Sci Biotechnol 5: 24 10.1186/2049-1891-5-24 24883184 PMC4025562

[17] Yu Z, Morrison M (2004) Improved extraction of PCR-quality community DNA from digesta and fecal samples. BioTechniques 36: 808–812. 15152600 10.2144/04365ST04

[18] Magoc T, Salzberg SL (2011) FLASH: fast length adjustment of short reads to improve genome assemblies. Bioinformatics 27: 2957–2963. 10.1093/bioinformatics/btr507 21903629 PMC3198573

[19] Edgar RC, Haas BJ, Clemente JC, Quince C, Knight R (2011) UCHIME improves sensitivity and speed of chimera detection. Bioinformatics 27: 2194–2200. 10.1093/bioinformatics/btr381 21700674 PMC3150044

[20] Caporaso JG, Kuczynski J, Stombaugh J, Bittinger K, Bushman FD, Costello EK, et al (2010) QIIME allows analysis of high-throughput community sequencing data. Nature Methods 7: 335–336. 10.1038/nmeth.f.303 20383131 PMC3156573

[21] Lozupone C, Knight R (2005) UniFrac: a new phylogenetic method for comparing microbial communities. Applied and Environmental Microbiology 71: 8228–8235. 16332807 10.1128/AEM.71.12.8228-8235.2005 PMC1317376

[22] Denman SE, McSweeney CS (2006) Development of a real-time PCR assay for monitoring anaerobic fungal and cellulolytic bacterial populations within the rumen. FEMS Microbiology Ecology 58: 572–582. 17117998 10.1111/j.1574-6941.2006.00190.x

[23] Denman SE, Tomkins N, McSweeney CS (2007) Quantitation and diversity analysis of ruminal methanogenic populations in response to the antimethanogenic compound bromochloromethane. Fems Microbiology Ecology 62: 313–322. 17949432 10.1111/j.1574-6941.2007.00394.x

[24] Weimer PJ, Stevenson DM, Mertens DR, Thomas EE (2008) Effect of monensin feeding and withdrawal on populations of individual bacterial species in the rumen of lactating dairy cows fed high-starch rations. Applied Microbiology and Biotechnology 80: 135–145. 10.1007/s00253-008-1528-9 18535825

[25] Stevenson DM, Weimer PJ (2007) Dominance of Prevotella and low abundance of classical ruminal bacterial species in the bovine rumen revealed by relative quantification real-time PCR. Applied Microbiology and Biotechnology 75: 165–174. 17235560 10.1007/s00253-006-0802-y

[26] Tymensen LD, McAllister TA (2012) Community Structure Analysis of Methanogens Associated with Rumen Protozoa Reveals Bias in Universal Archaeal Primers. Applied and Environmental Microbiology 78: 4051–4056. 10.1128/AEM.07994-11 22447586 PMC3346394

[27] Zhou ZM, Yu ZT, Meng QX (2012) Effects of nitrate on methane production, fermentation, and microbial populations in in vitro ruminal cultures. Bioresource Technology 103: 173–179. 10.1016/j.biortech.2011.10.013 22047657

[28] Mosoni P, Martin C, Forano E, Morgavi DP (2011) Long-term defaunation increases the abundance of cellulolytic ruminococci and methanogens but does not affect the bacterial and methanogen diversity in the rumen of sheep. Journal of Animal Science 89: 783–791. 10.2527/jas.2010-2947 21346137

[29] Patra AK, Saxena J (2010) A new perspective on the use of plant secondary metabolites to inhibit methanogenesis in the rumen. Phytochemistry 71: 1198–1222. 10.1016/j.phytochem.2010.05.010 20570294

[30] Hook SE, Northwood KS, Wright ADG, McBride BW (2009) Long-Term Monensin Supplementation Does Not Significantly Affect the Quantity or Diversity of Methanogens in the Rumen of the Lactating Dairy Cow. Applied and Environmental Microbiology 75: 374–380. 10.1128/AEM.01672-08 19028912 PMC2620707

[31] Hook SE, Steele MA, Northwood KS, Wright ADG, McBride BW (2011) Impact of High-Concentrate Feeding and Low Ruminal pH on Methanogens and Protozoa in the Rumen of Dairy Cows. Microbial Ecology 62: 94–105. 10.1007/s00248-011-9881-0 21625972

[32] Zhou M, Chung YH, Beauchemin KA, Holtshausen L, Oba M, McAllister TA, et al (2011) Relationship between rumen methanogens and methane production in dairy cows fed diets supplemented with a feed enzyme additive. Journal of Applied Microbiology 111: 1148–1158. 10.1111/j.1365-2672.2011.05126.x 21848695

[33] Castro-Carrera T, Toral PG, Frutos P, McEwan NR, Hervas G, Abecia L, et al (2014) Rumen bacterial community evaluated by 454 pyrosequencing and terminal restriction fragment length polymorphism analyses in dairy sheep fed marine algae. Journal of Dairy Science 97: 1661–1669. 10.3168/jds.2013-7243 24440247

[34] Jewell KA, McCormick C, Odt CL, Weimer PJ, Suen G (2015) Ruminal bacterial community composition in dairy cows is dynamic over the course of two lactations and correlates with feed efficiency. Appl Environ Microbiol. 10.1128/AEM.00720-15 PMC4551193 25934629

[35] Petri RM, Schwaiger T, Penner GB, Beauchemin KA, Forster RJ, McKinnon JJ, et al (2013) Characterization of the core rumen microbiome in cattle during transition from forage to concentrate as well as during and after an acidotic challenge. PLoS One 8: e83424 10.1371/journal.pone.0083424 24391765 PMC3877040

[36] Zhang RY, Zhu WY, Zhu W, Liu JX, Mao SY (2014) Effect of dietary forage sources on rumen microbiota, rumen fermentation and biogenic amines in dairy cows. Journal of the Science of Food and Agriculture 94: 1886–1895. 10.1002/jsfa.6508 24375419

[37] Jewell KA, McCormick CA, Odt CL, Weimer PJ, Suen G (2015) Ruminal Bacterial Community Composition in Dairy Cows Is Dynamic over the Course of Two Lactations and Correlates with Feed Efficiency. Applied and Environmental Microbiology 81: 4697–4710. 10.1128/AEM.00720-15 25934629 PMC4551193

[38] Singh KM, Jisha TK, Reddy B, Parmar N, Patel A, Patel AK, et al (2015) Microbial profiles of liquid and solid fraction associated biomaterial in buffalo rumen fed green and dry roughage diets by tagged 16S rRNA gene pyrosequencing. Molecular Biology Reports 42: 95–103. 10.1007/s11033-014-3746-9 25249226

[39] Wetzels SU, Mann E, Metzler-Zebeli BU, Wagner M, Klevenhusen F, Zebeli Q, et al (2015) Pyrosequencing reveals shifts in the bacterial epimural community relative to dietary concentrate amount in goats. Journal of Dairy Science 98: 5572–5587. 10.3168/jds.2014-9166 26051320

[40] Huo WJ, Zhu WY, Mao SY (2014) Impact of subacute ruminal acidosis on the diversity of liquid and solid-associated bacteria in the rumen of goats. World Journal of Microbiology & Biotechnology 30: 669–680. 24068532 10.1007/s11274-013-1489-8

[41] Purushe J, Fouts DE, Morrison M, White BA, Mackie RI, Coutinho PM, et al (2010) Comparative genome analysis of Prevotella ruminicola and Prevotella bryantii: insights into their environmental niche. Microbial Ecology 60: 721–729. 10.1007/s00248-010-9692-8 20585943

[42] Kobayashi Y, Shinkai T, Koike S (2008) Ecological and physiological characterization shows that Fibrobacter succinogenes is important in rumen fiber digestion—Review. Folia Microbiologica 53: 195–200. 10.1007/s12223-008-0024-z 18661290

[43] Kim M, Morrison M, Yu Z (2011) Status of the phylogenetic diversity census of ruminal microbiomes. FEMS Microbiol Ecol 76: 49–63. 10.1111/j.1574-6941.2010.01029.x 21223325

[44] Suen G, Weimer PJ, Stevenson DM, Aylward FO, Boyum J, Deneke J, et al (2011) The complete genome sequence of Fibrobacter succinogenes S85 reveals a cellulolytic and metabolic specialist. PLoS One 6: e18814 10.1371/journal.pone.0018814 21526192 PMC3079729

[45] Shinkai T, Ueki T, Kobayashi Y (2010) Detection and identification of rumen bacteria constituting a fibrolytic consortium dominated by Fibrobacter succinogenes. Anim Sci J 81: 72–79. 10.1111/j.1740-0929.2009.00698.x 20163675

[46] Ziolecki A, Wojciechowicz M (1980) Small Pectinolytic Spirochetes from the Rumen. Applied and Environmental Microbiology 39: 919–922. 7377780 10.1128/aem.39.4.919-922.1980 PMC291444

[47] Wojciechowicz M, Ziolecki A (1979) Pectinolytic Enzymes of Large Rumen Treponemes. Applied and Environmental Microbiology 37: 136–142. 32839 10.1128/aem.37.1.136-142.1979 PMC243413

[48] Janssen PH, Kirs M (2008) Structure of the archaeal community of the rumen. Applied and Environmental Microbiology 74: 3619–3625. 10.1128/AEM.02812-07 18424540 PMC2446570

[49] Popova M, Morgavi DP, Martin C (2013) Methanogens and Methanogenesis in the Rumens and Ceca of Lambs Fed Two Different High-Grain-Content Diets. Applied and Environmental Microbiology 79: 1777–1786. 10.1128/AEM.03115-12 23241983 PMC3592211

[50] Zhou M, Hernandez-Sanabria E, Guan LL (2009) Assessment of the Microbial Ecology of Ruminal Methanogens in Cattle with Different Feed Efficiencies. Applied and Environmental Microbiology 75: 6524–6533. 10.1128/AEM.02815-08 19717632 PMC2765141

[51] Wright ADG, Ma XL, Obispo NE (2008) Methanobrevibacter phylotypes are the dominant methanogens in sheep from Venezuela. Microbial Ecology 56: 390–394. 10.1007/s00248-007-9351-x 18165875

[52] Liu C, Zhu ZP, Liu YF, Guo TJ, Dong HM (2012) Diversity and abundance of the rumen and fecal methanogens in Altay sheep native to Xinjiang and the influence of diversity on methane emissions. Archives of Microbiology 194: 353–361. 10.1007/s00203-011-0757-y 22038025

