# Supplementary figures and images for: Effects of Diets Supplemented with Ensiled Mulberry Leaves and Sun-Dried Mulberry Fruit Pomace on the Ruminal Bacterial and Archaeal Community Composition of Finishing Steers
Source: PLoS One. 2016 Jun 3;11(6):e0156836. doi: 10.1371/journal.pone.0156836 (PMC4892645; doi:10.1371/journal.pone.0156836)

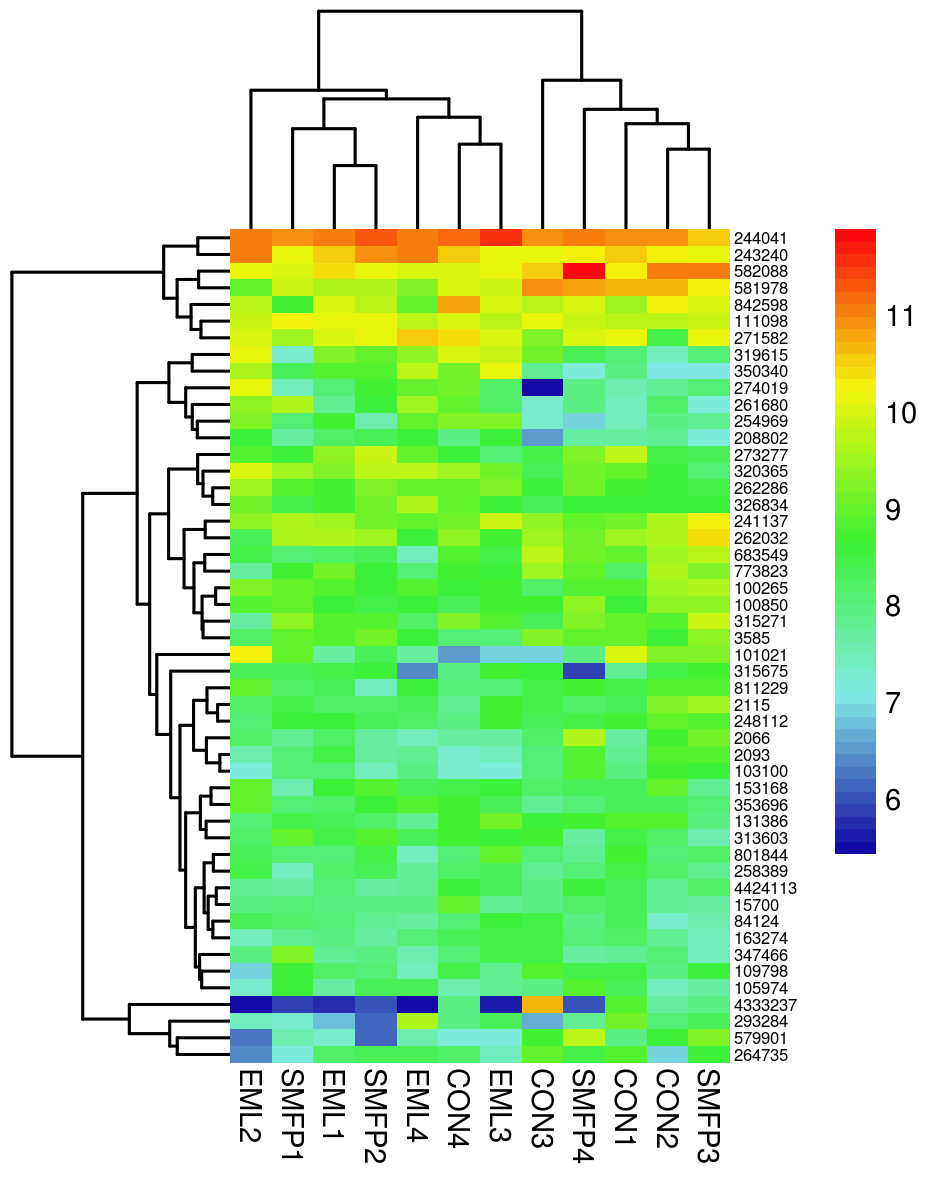

Supplement: S1 Fig — CON: control group (n = 4); EML: ensiled mulberry leaves group (n = 4); SMFP: sun-dried mulberry fruit pomace group (n = 4). (TIF) [file pone.0156836.s001.tif]
