# Supplementary material for: Effects of Diets Supplemented with Ensiled Mulberry Leaves and Sun-Dried Mulberry Fruit Pomace on the Ruminal Bacterial and Archaeal Community Composition of Finishing Steers
Source: PLoS One. 2016 Jun 3;11(6):e0156836. doi: 10.1371/journal.pone.0156836 (PMC4892645; doi:10.1371/journal.pone.0156836)
Supplement: S1 Table — (DOCX) [file pone.0156836.s002.docx]

Table S1. Individual finishing bulls for unique OTUs, richness estimates, and diversity indices within the rumen content.

| SampleID | SeqsNum | OTUsNum | EvenSeqsNum | EvenOTUsNum | ACE | simpson | shannon | PD_whole_tree | chao1 | observed_species | goods_coverage |
| --- | --- | --- | --- | --- | --- | --- | --- | --- | --- | --- | --- |
| CON1 | 165804 | 8775 | 100000 | 6922 | 12879.06 | 0.997363 | 10.21457 | 267.2937 | 12978.25 | 6922 | 0.96772 |
| CON2 | 237820 | 9568 | 100000 | 6402 | 11772.4 | 0.996961 | 10.02723 | 249.3974 | 11828.75 | 6402 | 0.97051 |
| CON3 | 257130 | 10350 | 100000 | 6698 | 12569.98 | 0.996909 | 10.0416 | 262.8374 | 12864.91 | 6698 | 0.96829 |
| CON4 | 226965 | 10311 | 100000 | 7114 | 12645.99 | 0.997024 | 10.21188 | 272.8816 | 12643.56 | 7114 | 0.96777 |
| EML1 | 218846 | 11261 | 100000 | 7706 | 14801.81 | 0.997493 | 10.35943 | 291.8359 | 14549.42 | 7706 | 0.96287 |
| EML2 | 198449 | 9362 | 100000 | 6871 | 12113.38 | 0.996833 | 10.09285 | 264.1438 | 12393.77 | 6871 | 0.96896 |
| EML3 | 197133 | 8844 | 100000 | 6487 | 11636.74 | 0.99693 | 10.1105 | 250.2677 | 12082.2 | 6487 | 0.97066 |
| EML4 | 206770 | 10241 | 100000 | 7314 | 13382.13 | 0.997241 | 10.28334 | 276.1805 | 13602.56 | 7314 | 0.96609 |
| SMFP1 | 214223 | 10322 | 100000 | 7227 | 13520.02 | 0.997635 | 10.31051 | 276.4773 | 14005.81 | 7227 | 0.96573 |
| SMFP2 | 210757 | 10080 | 100000 | 7145 | 12980.03 | 0.997159 | 10.25422 | 270.2439 | 13371.01 | 7145 | 0.9671 |
| SMFP3 | 178606 | 8067 | 100000 | 6178 | 10964.46 | 0.996735 | 9.925228 | 243.6522 | 11155.01 | 6178 | 0.97231 |
| SMFP4 | 202820 | 9145 | 100000 | 6560 | 12630.46 | 0.995882 | 9.937994 | 257.2607 | 12682.02 | 6560 | 0.9686 |

CON: control; EML: ensiled mulberry leaves; SMFP: sun-dried mulberry fruit pomace.
