# Supplementary material for: Effects of Diets Supplemented with Ensiled Mulberry Leaves and Sun-Dried Mulberry Fruit Pomace on the Ruminal Bacterial and Archaeal Community Composition of Finishing Steers
Source: PLoS One. 2016 Jun 3;11(6):e0156836. doi: 10.1371/journal.pone.0156836 (PMC4892645; doi:10.1371/journal.pone.0156836)
Supplement: S2 Table — (DOCX) [file pone.0156836.s003.docx]

Table S2. Relative abundance (%) of phylum in ruminal sample of individual finishing steers.

| Taxon | CON1 | CON2 | CON3 | CON4 | EML1 | EML2 | EML3 | EML4 | SMFP1 | SMFP2 | SMFP3 | SMFP4 |
| --- | --- | --- | --- | --- | --- | --- | --- | --- | --- | --- | --- | --- |
| k__Bacteria;p__Firmicutes | 46.633 | 40.976 | 47.058 | 44.939 | 43.956 | 40.236 | 46.012 | 42.361 | 47.808 | 47.241 | 36.42 | 43.191 |
| k__Bacteria;p__Bacteroidetes | 34.536 | 41.444 | 34.534 | 35.64 | 38.934 | 44.319 | 39.669 | 41.735 | 35.566 | 35.835 | 45.153 | 41.356 |
| k__Bacteria;p__Tenericutes | 5.778 | 3.044 | 4.736 | 6.191 | 4.209 | 2.762 | 3.395 | 4.413 | 4.773 | 4.714 | 3.134 | 4.583 |
| k__Bacteria;p__Proteobacteria | 2.649 | 2.538 | 3.146 | 1.861 | 1.97 | 2.633 | 1.439 | 1.551 | 1.879 | 1.37 | 2.026 | 1.368 |
| k__Bacteria;p__Verrucomicrobia | 2.034 | 2.942 | 1.674 | 1.897 | 2.077 | 1.867 | 1.565 | 1.915 | 1.582 | 1.676 | 2.583 | 1.165 |
| k__Bacteria;p__SR1 | 1.9 | 0.545 | 1.198 | 1.829 | 1.554 | 1.451 | 1.418 | 2.326 | 1.012 | 1.709 | 1.592 | 1.482 |
| k__Bacteria;p__Fibrobacteres | 1.882 | 1.771 | 2.124 | 1.572 | 2.093 | 0.892 | 0.995 | 1.344 | 2.241 | 2.242 | 2.629 | 1.681 |
| k__Bacteria;p__Spirochaetes | 1.295 | 1.905 | 1.957 | 1.457 | 1.574 | 1.427 | 1.171 | 0.849 | 1.657 | 1.771 | 1.739 | 1.729 |
| k__Archaea;p__Euryarchaeota | 1.213 | 1.966 | 1.322 | 2.325 | 1.613 | 1.69 | 1.717 | 0.99 | 0.713 | 1.527 | 1.718 | 1.512 |
| Unassigned;Other | 0.919 | 1.134 | 0.985 | 1.015 | 0.966 | 1.025 | 1.113 | 1.069 | 1.143 | 0.715 | 1.319 | 1.134 |
| k__Bacteria;p__Actinobacteria | 0.333 | 0.2 | 0.312 | 0.236 | 0.112 | 0.312 | 0.191 | 0.212 | 0.687 | 0.288 | 0.367 | 0.101 |
| k__Bacteria;p__Cyanobacteria | 0.181 | 0.395 | 0.132 | 0.07 | 0.161 | 0.27 | 0.093 | 0.156 | 0.262 | 0.126 | 0.454 | 0.063 |
| k__Bacteria;p__Lentisphaerae | 0.17 | 0.451 | 0.204 | 0.125 | 0.238 | 0.357 | 0.216 | 0.265 | 0.166 | 0.151 | 0.381 | 0.178 |
| k__Bacteria;p__Planctomycetes | 0.113 | 0.202 | 0.148 | 0.162 | 0.106 | 0.179 | 0.185 | 0.139 | 0.117 | 0.118 | 0.102 | 0.106 |
| k__Bacteria;p__Chloroflexi | 0.112 | 0.075 | 0.084 | 0.173 | 0.14 | 0.15 | 0.224 | 0.202 | 0.109 | 0.149 | 0.088 | 0.072 |
| k__Bacteria;p__Elusimicrobia | 0.091 | 0.188 | 0.144 | 0.094 | 0.075 | 0.088 | 0.349 | 0.118 | 0.09 | 0.115 | 0.132 | 0.117 |
| k__Bacteria;p__Armatimonadetes | 0.041 | 0.027 | 0.039 | 0.117 | 0.04 | 0.082 | 0.079 | 0.114 | 0.042 | 0.051 | 0.032 | 0.058 |
| k__Bacteria;p__WPS-2 | 0.034 | 0.059 | 0.072 | 0.093 | 0.058 | 0.111 | 0.049 | 0.09 | 0.05 | 0.058 | 0.044 | 0.032 |
| k__Bacteria;p__Synergistetes | 0.025 | 0.068 | 0.027 | 0.055 | 0.033 | 0.044 | 0.045 | 0.051 | 0.021 | 0.049 | 0.035 | 0.02 |
| k__Bacteria;p__LD1 | 0.022 | 0.051 | 0.026 | 0.031 | 0.027 | 0.042 | 0.054 | 0.076 | 0.029 | 0.045 | 0.037 | 0.019 |
| k__Bacteria;p__TM7 | 0.016 | 0.008 | 0.061 | 0.065 | 0.052 | 0.034 | 0.01 | 0.014 | 0.03 | 0.04 | 0.004 | 0.021 |
| k__Bacteria;p__Acidobacteria | 0.015 | 0.004 | 0.001 | 0.038 | 0.005 | 0.013 | 0.005 | 0.005 | 0.005 | 0.001 | 0.003 | 0.002 |
| k__Bacteria;p__Fusobacteria | 0.004 | 0.004 | 0.006 | 0.008 | 0.004 | 0.011 | 0.002 | 0.002 | 0.003 | 0.003 | 0.005 | 0.007 |
| k__Archaea;p__Crenarchaeota | 0.001 | 0 | 0.001 | 0.002 | 0 | 0.001 | 0 | 0 | 0.006 | 0 | 0.001 | 0.001 |
| k__Bacteria;p__AD3 | 0.001 | 0 | 0.002 | 0 | 0 | 0.001 | 0.001 | 0 | 0 | 0 | 0 | 0 |
| k__Bacteria;p__Nitrospirae | 0.001 | 0.001 | 0 | 0.001 | 0.001 | 0 | 0.001 | 0 | 0 | 0 | 0 | 0 |
| k__Bacteria;p__[Thermi] | 0.001 | 0 | 0.006 | 0 | 0.001 | 0 | 0 | 0 | 0 | 0.002 | 0.001 | 0 |
| k__Bacteria;Other | 0 | 0.001 | 0 | 0 | 0 | 0.001 | 0 | 0.001 | 0 | 0 | 0.001 | 0 |
| k__Bacteria;p__ | 0 | 0 | 0 | 0.001 | 0 | 0 | 0 | 0 | 0 | 0 | 0 | 0.001 |
| k__Bacteria;p__BRC1 | 0 | 0 | 0 | 0 | 0 | 0 | 0.001 | 0 | 0 | 0.002 | 0 | 0 |
| k__Bacteria;p__Chlamydiae | 0 | 0 | 0 | 0 | 0 | 0.001 | 0 | 0.001 | 0.001 | 0 | 0 | 0 |
| k__Bacteria;p__GN02 | 0 | 0 | 0 | 0 | 0 | 0 | 0 | 0.001 | 0 | 0.002 | 0 | 0.001 |
| k__Bacteria;p__Gemmatimonadetes | 0 | 0 | 0 | 0.002 | 0.001 | 0 | 0 | 0 | 0.005 | 0 | 0 | 0 |
| k__Bacteria;p__NKB19 | 0 | 0 | 0 | 0 | 0 | 0 | 0.001 | 0 | 0 | 0 | 0 | 0 |
| k__Bacteria;p__OP11 | 0 | 0 | 0.001 | 0 | 0 | 0.001 | 0 | 0 | 0.002 | 0 | 0 | 0 |
| k__Bacteria;p__WS3 | 0 | 0.001 | 0 | 0.001 | 0 | 0 | 0 | 0 | 0.001 | 0 | 0 | 0 |

CON: control; EML: ensiled mulberry leaves; SMFP: sun-dried mulberry fruit pomace.
