# Supplementary material for: Effects of Diets Supplemented with Ensiled Mulberry Leaves and Sun-Dried Mulberry Fruit Pomace on the Ruminal Bacterial and Archaeal Community Composition of Finishing Steers
Source: PLoS One. 2016 Jun 3;11(6):e0156836. doi: 10.1371/journal.pone.0156836 (PMC4892645; doi:10.1371/journal.pone.0156836)
Supplement: S3 Table — (DOCX) [file pone.0156836.s004.docx]

Table S3. Relative abundance (%) of shared genus in ruminal sample of individual finishing steers.

| Taxon | CON1 | CON2 | CON3 | CON4 | EML1 | EML2 | EML3 | EML4 | SMFP1 | SMFP2 | SMFP3 | SMFP4 |
| --- | --- | --- | --- | --- | --- | --- | --- | --- | --- | --- | --- | --- |
| g__Prevotella | 12.085 | 18.83 | 12.845 | 9.597 | 13.249 | 12.211 | 12.305 | 11.266 | 12.755 | 10.339 | 22.066 | 18.329 |
| g__Ruminococcus | 2.706 | 2.582 | 2.976 | 1.829 | 2.63 | 2.102 | 2.271 | 2.191 | 2.625 | 2.927 | 2.465 | 2.851 |
| g__Succiniclasticum | 2.832 | 2.533 | 3.224 | 2.23 | 1.781 | 1.856 | 2.418 | 1.676 | 1.989 | 1.742 | 2.04 | 2.838 |
| g__Butyrivibrio | 2.68 | 1.885 | 2.435 | 1.745 | 2.083 | 2.597 | 2.279 | 2.436 | 2.684 | 3.018 | 1.73 | 3.005 |
| g__Fibrobacter | 1.882 | 1.771 | 2.124 | 1.572 | 2.093 | 0.892 | 0.995 | 1.344 | 2.241 | 2.242 | 2.629 | 1.681 |
| g__Methanobrevibacter | 1.097 | 1.77 | 1.204 | 2.196 | 1.481 | 1.507 | 1.511 | 0.852 | 0.604 | 1.405 | 1.625 | 1.439 |
| g__Treponema | 1.213 | 1.768 | 1.882 | 1.356 | 1.434 | 1.189 | 1.023 | 0.672 | 1.479 | 1.646 | 1.656 | 1.645 |
| g__YRC22 | 1.982 | 1.678 | 1.18 | 1.517 | 1.832 | 0.778 | 1.298 | 1.201 | 2.143 | 1.443 | 2.385 | 2.224 |
| g__Anaerovibrio | 0.387 | 1.322 | 1.081 | 0.577 | 0.752 | 0.334 | 0.559 | 0.394 | 0.651 | 0.566 | 1.026 | 0.752 |
| g__CF231 | 1.045 | 1.268 | 1.115 | 1.248 | 1.512 | 1.137 | 1.567 | 1.396 | 1.19 | 1.346 | 1.678 | 1.134 |
| g__Coprococcus | 1.347 | 0.879 | 1.229 | 1.125 | 1.18 | 0.72 | 0.847 | 0.999 | 1.601 | 1.294 | 1.104 | 1.327 |
| g__RFN20 | 0.427 | 0.61 | 0.664 | 0.726 | 0.529 | 0.453 | 0.731 | 0.568 | 0.35 | 0.486 | 0.548 | 0.547 |
| g__Clostridium | 0.857 | 0.589 | 0.939 | 0.87 | 0.916 | 0.58 | 0.71 | 0.756 | 1.25 | 0.955 | 0.621 | 0.767 |
| g__BF311 | 0.31 | 0.392 | 0.351 | 0.355 | 0.408 | 0.711 | 0.451 | 0.445 | 0.411 | 0.332 | 0.328 | 0.251 |
| g__Anaeroplasma | 0.353 | 0.313 | 0.267 | 0.254 | 0.255 | 0.163 | 0.352 | 0.208 | 0.429 | 0.285 | 0.343 | 0.633 |
| g__Succinivibrio | 0.026 | 0.271 | 0.006 | 0.003 | 0.005 | 0.006 | 0.001 | 0.004 | 0.013 | 0.003 | 0.036 | 0.005 |
| g__Desulfovibrio | 0.194 | 0.243 | 0.221 | 0.231 | 0.2 | 0.169 | 0.26 | 0.228 | 0.168 | 0.228 | 0.179 | 0.165 |
| g__Acetobacter | 0.464 | 0.21 | 1.676 | 0.256 | 0.057 | 0.051 | 0.052 | 0.046 | 0.067 | 0.063 | 0.283 | 0.073 |
| g__Oscillospira | 0.15 | 0.204 | 0.164 | 0.156 | 0.135 | 0.138 | 0.252 | 0.17 | 0.125 | 0.149 | 0.105 | 0.157 |
| g__Moryella | 0.203 | 0.196 | 0.176 | 0.223 | 0.167 | 0.15 | 0.192 | 0.205 | 0.145 | 0.218 | 0.252 | 0.26 |
| g__Anaerostipes | 0.215 | 0.188 | 0.25 | 0.248 | 0.238 | 0.215 | 0.171 | 0.229 | 0.564 | 0.23 | 0.481 | 0.501 |
| g__Mogibacterium | 0.213 | 0.174 | 0.161 | 0.188 | 0.186 | 0.222 | 0.243 | 0.18 | 0.161 | 0.23 | 0.124 | 0.176 |
| g__Pseudomonas | 0.123 | 0.139 | 0.167 | 0.122 | 0.096 | 0.079 | 0.157 | 0.088 | 0.15 | 0.117 | 0.124 | 0.127 |
| g__Shuttleworthia | 0.111 | 0.137 | 0.156 | 0.207 | 0.154 | 0.119 | 0.246 | 0.118 | 0.137 | 0.129 | 0.088 | 0.119 |
| g__Lactobacillus | 0.352 | 0.108 | 0.703 | 0.34 | 0.1 | 0.218 | 0.063 | 0.148 | 0.141 | 0.122 | 0.179 | 0.073 |
| g__Methanosphaera | 0.061 | 0.091 | 0.046 | 0.058 | 0.037 | 0.047 | 0.096 | 0.038 | 0.032 | 0.038 | 0.037 | 0.034 |
| g__p-75-a5 | 0.086 | 0.088 | 0.107 | 0.144 | 0.083 | 0.102 | 0.1 | 0.121 | 0.114 | 0.132 | 0.083 | 0.097 |
| g__SHD-231 | 0.112 | 0.074 | 0.082 | 0.169 | 0.139 | 0.145 | 0.223 | 0.202 | 0.108 | 0.149 | 0.088 | 0.071 |
| g__Dehalobacterium | 0.061 | 0.071 | 0.041 | 0.105 | 0.068 | 0.138 | 0.135 | 0.142 | 0.06 | 0.096 | 0.045 | 0.054 |
| g__Blautia | 0.076 | 0.061 | 0.052 | 0.06 | 0.073 | 0.053 | 0.08 | 0.04 | 0.05 | 0.07 | 0.042 | 0.052 |
| g__L7A_E11 | 0.069 | 0.059 | 0.055 | 0.095 | 0.079 | 0.076 | 0.064 | 0.055 | 0.064 | 0.078 | 0.067 | 0.083 |
| g__Corynebacterium | 0.058 | 0.045 | 0.058 | 0.071 | 0.04 | 0.088 | 0.065 | 0.075 | 0.068 | 0.058 | 0.045 | 0.025 |
| g__TG5 | 0.009 | 0.038 | 0.011 | 0.041 | 0.018 | 0.031 | 0.033 | 0.03 | 0.011 | 0.033 | 0.009 | 0.004 |
| g__Ruminobacter | 0.02 | 0.03 | 0.011 | 0.01 | 0.008 | 0.246 | 0.003 | 0.007 | 0.056 | 0.014 | 0.013 | 0.027 |
| g__[Prevotella] | 0.034 | 0.03 | 0.033 | 0.022 | 0.03 | 0.008 | 0.033 | 0.024 | 0.039 | 0.025 | 0.048 | 0.043 |
| g__Bulleidia | 0.035 | 0.026 | 0.041 | 0.037 | 0.037 | 0.025 | 0.036 | 0.036 | 0.047 | 0.049 | 0.023 | 0.06 |
| g__Acinetobacter | 0.018 | 0.024 | 0.018 | 0.157 | 0.003 | 0.005 | 0.01 | 0.012 | 0.014 | 0.012 | 0.019 | 0.01 |
| g__vadinCA11 | 0.016 | 0.023 | 0.007 | 0.024 | 0.036 | 0.04 | 0.037 | 0.046 | 0.028 | 0.016 | 0.027 | 0.012 |
| g__Bacillus | 0.04 | 0.023 | 0.036 | 0.026 | 0.018 | 0.019 | 0.03 | 0.016 | 0.021 | 0.013 | 0.018 | 0.012 |
| g__Pyramidobacter | 0.009 | 0.022 | 0.007 | 0.01 | 0.01 | 0.009 | 0.006 | 0.013 | 0.007 | 0.008 | 0.024 | 0.007 |
| g__Bilophila | 0.038 | 0.022 | 0.035 | 0.041 | 0.026 | 0.029 | 0.036 | 0.038 | 0.027 | 0.036 | 0.024 | 0.023 |
| g__Paludibacter | 0.095 | 0.02 | 0.012 | 0.041 | 0.027 | 0.054 | 0.036 | 0.065 | 0.026 | 0.014 | 0.016 | 0.023 |
| g__Pseudobutyrivibrio | 0.027 | 0.015 | 0.042 | 0.031 | 0.04 | 0.017 | 0.032 | 0.034 | 0.058 | 0.037 | 0.027 | 0.052 |
| g__Comamonas | 0.006 | 0.012 | 0.02 | 0.044 | 0.008 | 0.013 | 0.002 | 0.004 | 0.008 | 0.003 | 0.024 | 0.002 |
| g__Adlercreutzia | 0.011 | 0.008 | 0.008 | 0.008 | 0.005 | 0.007 | 0.02 | 0.01 | 0.004 | 0.011 | 0.007 | 0.009 |
| g__Rummeliibacillus | 0.002 | 0.007 | 0.009 | 0.01 | 0.004 | 0.004 | 0.006 | 0.007 | 0.005 | 0.008 | 0.005 | 0.003 |
| g__Atopobium | 0.003 | 0.007 | 0.006 | 0.004 | 0.003 | 0.008 | 0.005 | 0.002 | 0.001 | 0.005 | 0.004 | 0.002 |
| g__Syntrophomonas | 0.007 | 0.007 | 0.002 | 0.004 | 0.009 | 0.011 | 0.011 | 0.012 | 0.008 | 0.01 | 0.008 | 0.007 |
| g__Paenibacillus | 0.011 | 0.007 | 0.006 | 0.004 | 0.005 | 0.002 | 0.004 | 0.002 | 0.003 | 0.003 | 0.001 | 0.002 |
| g__Anaerofustis | 0.01 | 0.005 | 0.007 | 0.01 | 0.008 | 0.039 | 0.01 | 0.014 | 0.005 | 0.016 | 0.008 | 0.011 |
| g__Cetobacterium | 0.002 | 0.004 | 0.006 | 0.008 | 0.002 | 0.01 | 0.001 | 0.002 | 0.002 | 0.003 | 0.003 | 0.007 |
| g__Geobacillus | 0.007 | 0.004 | 0.006 | 0.003 | 0.001 | 0.008 | 0.005 | 0.004 | 0.005 | 0.004 | 0.004 | 0.005 |
| g__[Ruminococcus] | 0.006 | 0.003 | 0.005 | 0.003 | 0.002 | 0.004 | 0.003 | 0.002 | 0.001 | 0.009 | 0.001 | 0.001 |
| g__PSB-M-3 | 0.003 | 0.002 | 0.002 | 0.005 | 0.011 | 0.002 | 0.004 | 0.017 | 0.005 | 0.002 | 0.001 | 0.005 |
| g__Paracoccus | 0.004 | 0.002 | 0.003 | 0.001 | 0.001 | 0.001 | 0.001 | 0.004 | 0.002 | 0.002 | 0.002 | 0.001 |
| g__Brevibacillus | 0.005 | 0.002 | 0.001 | 0.004 | 0.003 | 0.001 | 0.003 | 0.002 | 0.003 | 0.004 | 0.003 | 0.002 |
| g__Dorea | 0.006 | 0.002 | 0.002 | 0.014 | 0.004 | 0.015 | 0.01 | 0.001 | 0.002 | 0.002 | 0.003 | 0.002 |
| g__Lactococcus | 0.008 | 0.002 | 0.012 | 0.003 | 0.001 | 0.008 | 0.001 | 0.005 | 0.004 | 0.008 | 0.005 | 0.004 |

CON: control; EML: ensiled mulberry leaves; SMFP: sun-dried mulberry fruit pomace.
