# Supplementary material for: Effects of Diets Supplemented with Ensiled Mulberry Leaves and Sun-Dried Mulberry Fruit Pomace on the Ruminal Bacterial and Archaeal Community Composition of Finishing Steers
Source: PLoS One. 2016 Jun 3;11(6):e0156836. doi: 10.1371/journal.pone.0156836 (PMC4892645; doi:10.1371/journal.pone.0156836)
Supplement: S5 Table — (DOCX) [file pone.0156836.s006.docx]

Table S5. Primers used in real-time PCR for the detection of ruminal archaeal and bacterial species/taxa

| Target taxon and specific strain tested | | Primer set | Primer sequences |  |
| --- | --- | --- | --- | --- |
| Bacteria |  |  |  |  |
|  | Total bacteria | BacteriaR  BacteriaF | CGGCAACGAGCGCAACCC  CCATTGTAGCACGTGTGTAGCC |  |
|  | *Butyrivibrio fibrisolvens H17c* | ButFib2F  ButFib2R | ACCGCATAAGCGCACGGA  CGGGTCCATCTTGTACCGATAAAT |  |
|  | *Eubacterium ruminantium GA195* | EubRum2F  EubRum2R | CTCCCGAGACTGAGGAAGCTTG  GTCCATCTCACACCACCGGA |  |
|  | *Fibrobacter succinogenes S85* | FibSuc3F  FibSuc3R | GCGGGTAGCAAACAGGATTAGA  CCCCCGGACACCCAGTAT |  |
|  | *Megasphaera elsdenil T81* | MegEls2F  MegEls2R | AGATGGGGACAACAGCTGGA  CGAAAGCTCCGAAGAGCCT |  |
|  | *Prevotella brevis B_1_4* | PreBre1F  PreBre1R | GGTTTCCTTGAGTGTATTCGACGTC  CTTTCGCTTGGCCGCTG |  |
|  | *Prevotella bryantii GA33* | PreBry2F  PreBry2R | AGCGCAGGCCGTTTGG  GCTTCCTGTGCACTCAAGTCTGAC |  |
|  | *Prevotella ruminicola 23* | PreRum1F  PreRum1R | GAAAGTCGGATTAATGCTCTATGTT  CATCCTATAGCGGTAAACCTTTGG |  |
|  | *Ruminobacter amylophilus H18* | RmbAmy2F  RmbAmy2R | CTGGGGAGCTGCCTGAATG  GCATCTGAATGCGACTGGTTG |  |
|  | *Ruminococcus albus 7* | RumAlb3F  RumAlb3R | TGTTAACAGAGGGAAGCAAAGCA  TGCAGCCTACAATCCGAACTAA |  |
|  | *Ruminococcus flavefaciens FD-1* | RumFla3F  RumFla3R | TGGCGGACGGGTGAGTAA  TTACCATCCGTTTCCAGAAGCT |  |
|  | *Selenomonas ruminantium D* | SelRum2F  SelRum2R | CAATAAGCATTCCGCCTGGG  TTCACTCAATGTCAAGCCCTGG |  |
|  | *Streptococcus bovis JB1* | StrBov2F  StrBov2R | TTCCTAGAGATAGGAAGTTTCTTCGG  ATGATGGCAACTAACAATAGGGGT |  |
|  | *Succinivibrio dextrinosolvens 22b* | SucDex1F  SucDex1R | CGTCAGCTCGTGTCGTGAGA  CCCGCTGGCAACAAAGG |  |
|  |  |  |  |  |
|  |  |  |  |  |
| Archaea |  |  |  |  |
|  | Methanogen | MethanogenF  MethanogenR | GGATTAGATACCCSGGTAGT  GTTGARTCCAATTAAACCGCA |  |
|  | Methanogen | Met86F  Met915R | GCTCAGTAACACGTGG  GTGCTCCCCCGCCAATTCCT |  |
|  | Methanogen | NestmetF  NestmetR | AMGWTCCAGGCCCTACGG  TGGCACCSGTCTTRCCC |  |
|  | *Methanobrevibacter* spp. | NestMbbF  NestMbbR | TGGGAATTGCTGGWGATACTRTT  GGAGCRGCTCAAAGCCA |  |
|  | *Methanomicrobium* spp. | NestMmF  NestMmR | TGTTTAAAACACATGGGAAGA  ATTCCCAGTATCTCTTAGACGC |  |
|  | RCC | NestRCCR  NestRCCF | TTCTGGGGTAGGGGTAAAATC  GTCTGCAGCGTTTACACCCT |  |
